# Supplementary figures and images for: Genetic diversity and demographic history of the largest remaining migratory population of brindled wildebeest (Connochaetes taurinus taurinus) in southern Africa
Source: PLoS One. 2025 Apr 24;20(4):e0310580. doi: 10.1371/journal.pone.0310580 (PMC12021205; doi:10.1371/journal.pone.0310580)

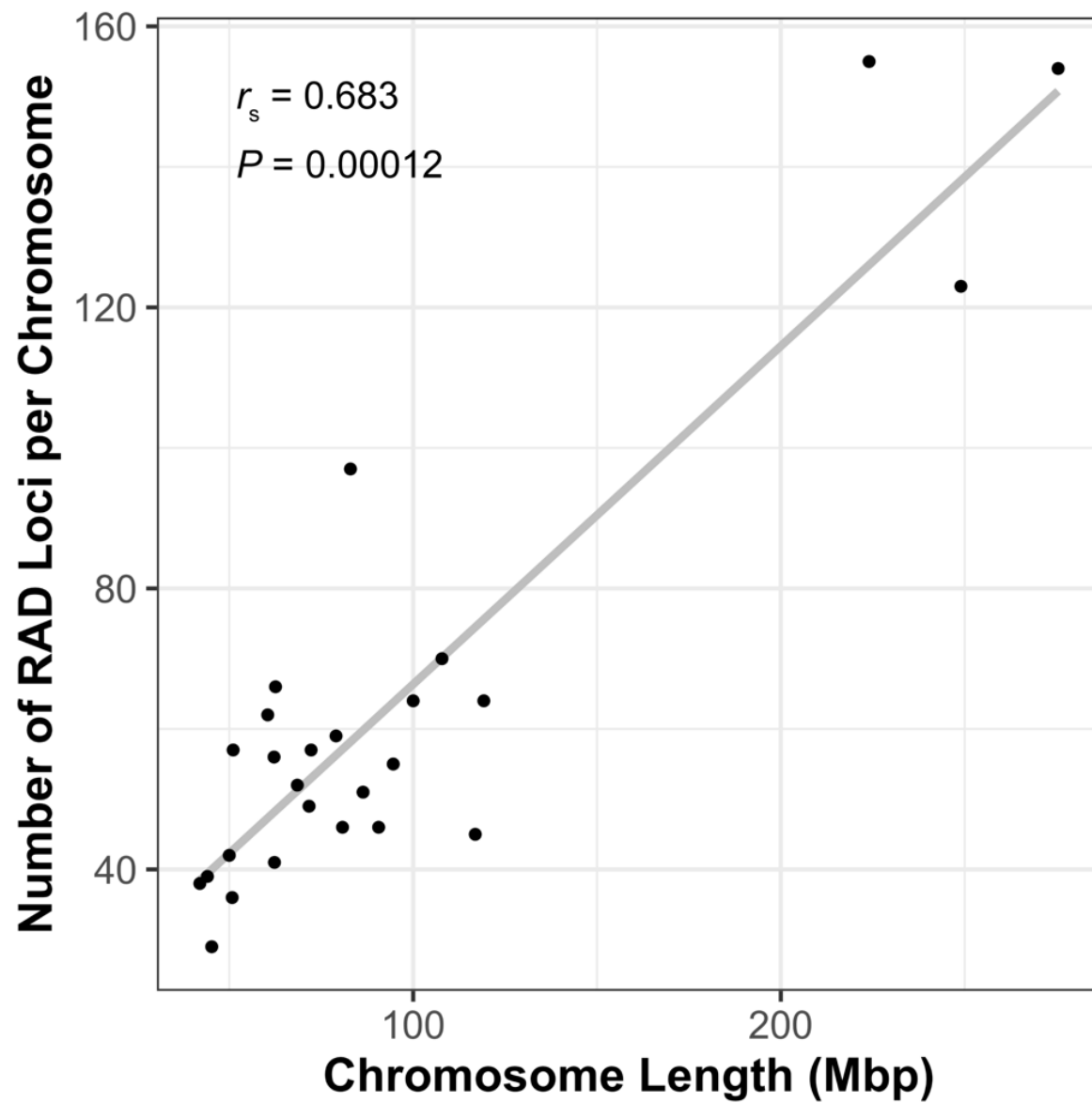

Supplement: S1 Fig — The linear regression line is shown in grey. The number of RAD loci mapping to a chromosome was significantly correlated with chromosome length (Spearman’s ρ = 0.683, P = 0.00012). (PDF) [file pone.0310580.s001.pdf]

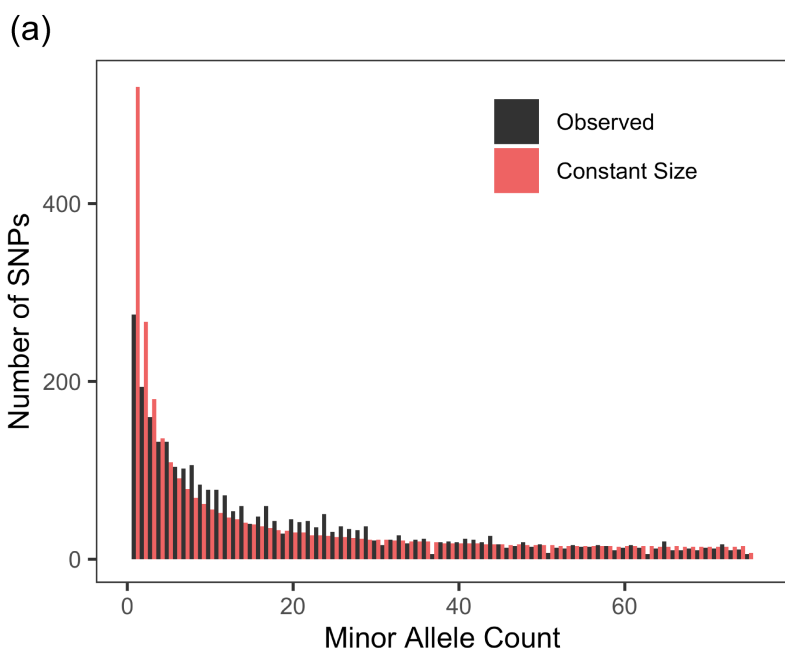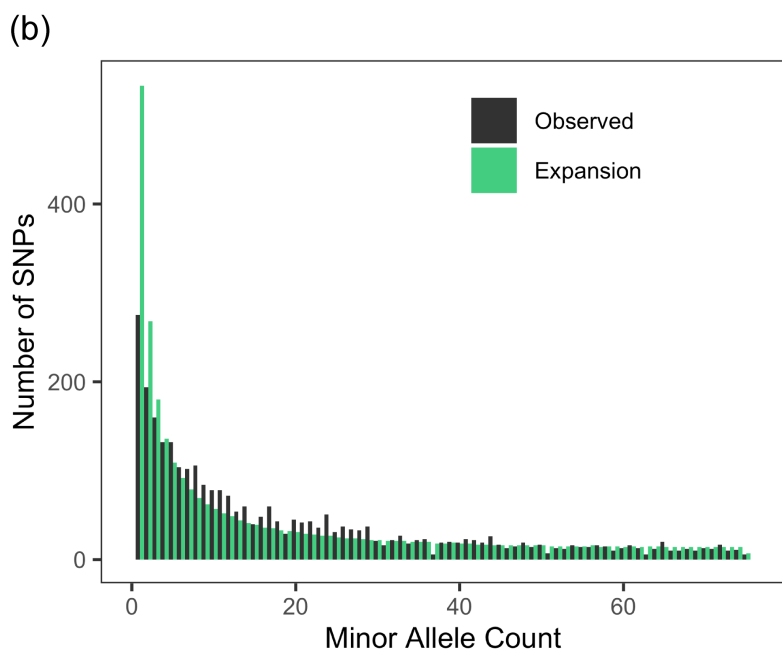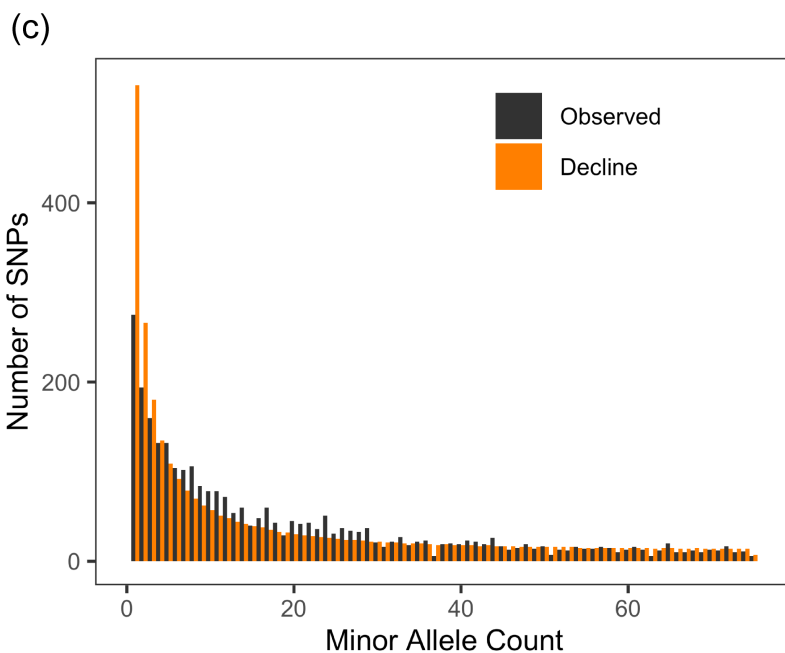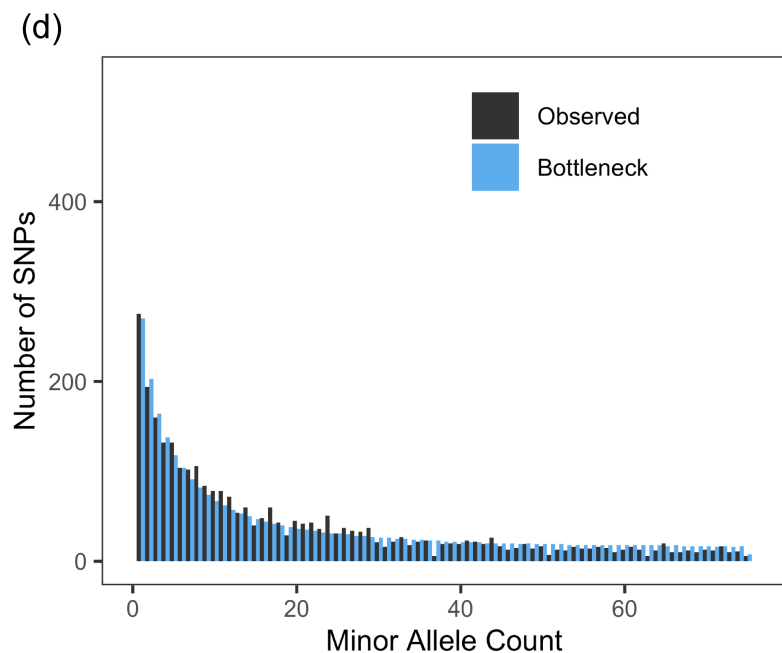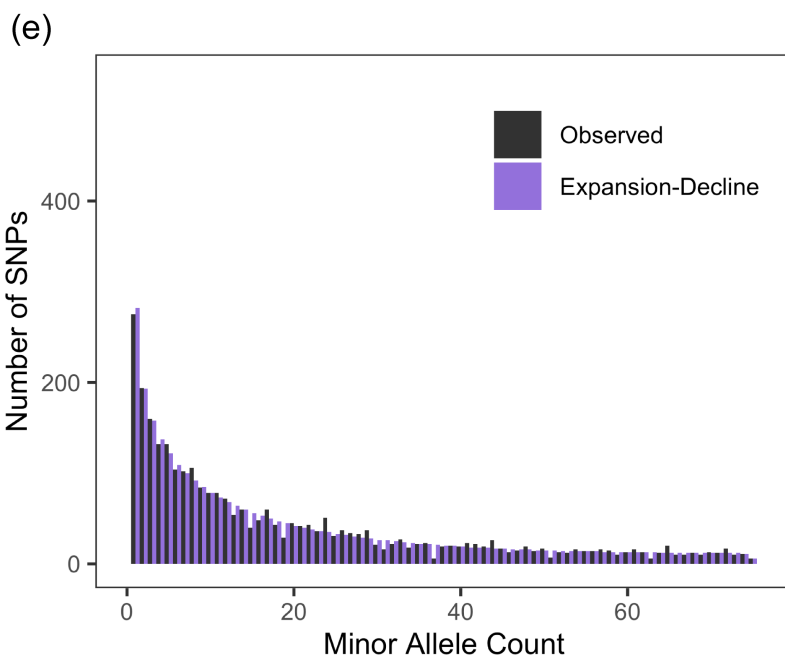

Supplement: S3 Fig — (a) Constant population size, (b) population expansion, (c) population decline, (d) bottleneck, (e) population expansion followed by decline. (PDF) [file pone.0310580.s003.pdf]
