# Supplementary material for: Genetic diversity and demographic history of the largest remaining migratory population of brindled wildebeest (Connochaetes taurinus taurinus) in southern Africa
Source: PLoS One. 2025 Apr 24;20(4):e0310580. doi: 10.1371/journal.pone.0310580 (PMC12021205; doi:10.1371/journal.pone.0310580)
Supplement: S2 Fig — None of these K values had a higher likelihood than K = 1, and the assignment of most individuals to multiple clusters indicates that there is no genetic population structure within the GLE. (PDF) [file pone.0310580.s002.pdf]

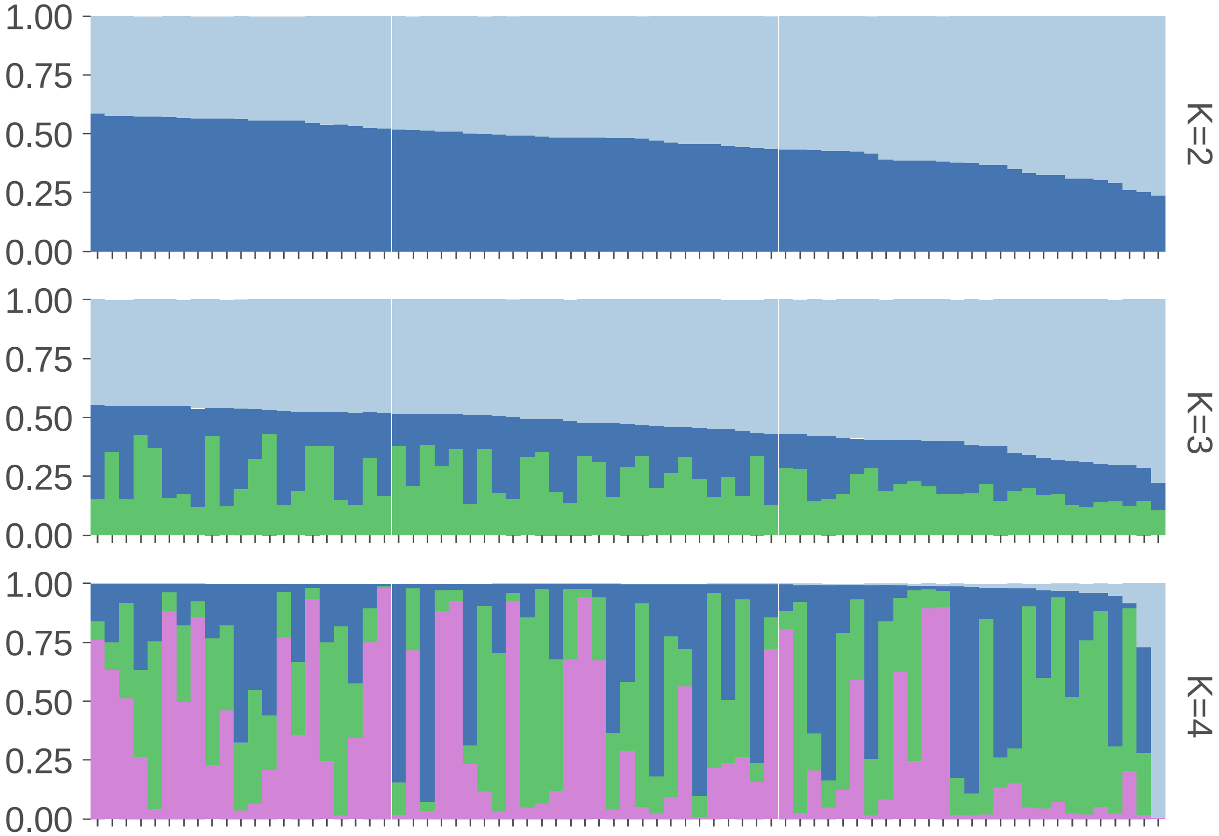

Figure S2. Structure plots for 75 wildebeest from the Greater Liuwa Ecosystem based on 1,730 SNPs assuming *K*=2, *K*=3, and *K*=4 clusters. None of these *K* values had a higher likelihood than *K*=1, and the assignment of most individuals to multiple clusters indicates that there is no genetic population structure within the GLE.
