# Supplementary material for: Genetic diversity and demographic history of the largest remaining migratory population of brindled wildebeest (Connochaetes taurinus taurinus) in southern Africa
Source: PLoS One. 2025 Apr 24;20(4):e0310580. doi: 10.1371/journal.pone.0310580 (PMC12021205; doi:10.1371/journal.pone.0310580)
Supplement: S1 Table — Data is presented separately for loci aligned to the domestic sheep (Ovis aries) or brindled wildebeest (Connochaetes taurinus) reference genomes. Loci aligned to the wildebeest genome were used for downstream analyses. (PDF) [file pone.0310580.s004.pdf]

Table S1. Number of putative RAD loci remaining after each step of the quality filtering protocol. Data is presented separately for loci aligned to the domestic sheep (*Ovis aries*) or blue wildebeest (*Connochaetes taurinus*) reference genomes. Loci aligned to the wildebeest genome were used for downstream analyses.

Step	Filtering requirement	Aligned to sheep reference genome	Aligned to wildebeest reference genome
1	Total putative loci	19,437	19,459
2	Contains ≥ 1 SNP	12,404	12,430
3	Genotyped in ≥ 90% of samples	3,413	3,415
4	< 5% genotypes with three alleles or a bad het. ratio	2,986	2,986
5	No loci with significant heterozygote excess	2,967	2,965
6	No high read depth outliers	2,966	2,964
7	One high-quality BLAST hit to reference genome[a]	2,190	2,275
8	No loci with BLAST hit to X-chromosome	2,159	2,244
9	No loci with significant homozygote excess	2,096	2,160
10	Meets expectations of infinite sites model	2,017	2,027
11	Problem loci identified through manual checks removed	1,956	1,964
12	Loci with ≤ 4 SNPs	1,921	1,879
13	Loci with no singleton SNPs (MAF > 0.01)	**1,773**	**1,730**

[a]23 loci produced no hits to either the sheep or wildebeest genomes, but blastn searches resulted in hits to the genomes of related bovid taxa.
