# Supplementary material for: Genetic diversity and demographic history of the largest remaining migratory population of brindled wildebeest (Connochaetes taurinus taurinus) in southern Africa
Source: PLoS One. 2025 Apr 24;20(4):e0310580. doi: 10.1371/journal.pone.0310580 (PMC12021205; doi:10.1371/journal.pone.0310580)
Supplement: S2 Table — For each K value (number of genetic clusters assumed in the model), we report the number of replicates, mean and standard deviation log probability of the data given the number of clusters, and Evanno statistic, ΔK. (PDF) [file pone.0310580.s005.pdf]

Table S2. Statistics obtained from Structure Harvester used to determine the optimal number of genetic clusters for wildebeest within the Greater Liuwa Ecosystem. For each *K* value (number of genetic clusters assumed in the model), we report the number of replicates, mean and standard deviation log probability of the data given the number of clusters, and Evanno statistic, Δ*K*.

K	**Reps**	**Mean LnP(*K*)**	**SD LnP(*K*)**	**Δ*K***
1	10	-87701.38	3.19	NA
2	10	-94607.62	4652.74	1.28
3	10	-95549.65	13257.36	0.51
4	10	-89766.43	1509.91	NA
