# Supplementary material for: Genetic diversity and demographic history of the largest remaining migratory population of brindled wildebeest (Connochaetes taurinus taurinus) in southern Africa
Source: PLoS One. 2025 Apr 24;20(4):e0310580. doi: 10.1371/journal.pone.0310580 (PMC12021205; doi:10.1371/journal.pone.0310580)
Supplement: S3 Table — Parameters describe effective population size (N) or the time a demographic event occurred (T) in years before present. Estimates have been rounded to the nearest hundred. The model with the highest support is shown in bold. (PDF) [file pone.0310580.s006.pdf]

**Table S3. Parameter estimates from the highest likelihood model for each demographic scenario tested in fastsimcoal2.** Parameters describe effective population size (*N*) or the time a demographic event occurred (*T*) in years before present. Estimates have been rounded to the nearest hundred. The model with the highest support is shown in bold.

| **Model** | $N_{current}$ | $N_{ancestral}$ | $N_{intermediate}$ | $T_{expansion}$ | $T_{decline}$ |
|---|---|---|---|---|---|
| Constant size | 45,200 | - | - | - | - |
| Expansion | 44,100 | 41,900 | - | 1,501,300 | - |
| Decline | 9,800 | 53,400 | - | - | 4,000 |
| Bottleneck | 5,300 | 52,900 | 4,400 | - | 1,000 |
| **Expansion-Decline** | **13,400** | **26,800** | **92,700** | **635,600** | **15,480** |
